# Epistemic Arguments For A Democratic Right to Silence

**DOI:** 10.1093/pq/pqad128

**Published:** 2024-01-18

**Authors:** Dan Degerman, Francesca Bellazzi

**Affiliations:** 1 Department of Philosophy, University of Bristol, UK; 2 Department of Philosophy, University of Birmingham, UK; Department of Philosophy, University of Bristol, UK

**Keywords:** silence, rights, epistemology, epistemic injustice, democracy, lying

## Abstract

While much ink has been spilt over the political importance of speech, much less has been dedicated to the political importance of silence. This article seeks to fill that gap. We propose the need for a robust, democratic right to silence in public life and argue that there are politically salient epistemic reasons for recognising that right. We begin by defining what silence is and what a robust right to silence entails. We then argue that the right to silence offers two politically salient epistemic benefits. The first is that, if the right to silence is maintained, we can avoid the epistemic harm that may be caused when an individual is compelled to lie in public. The second is that the right to silence can protect marginalised individuals against the epistemic injustices that may arise when others are likely to misconstrue their speech.

Creon: If silence is not allowed, what is anyone allowed?

(Seneca, Oedipus. In: *Tragedies*, Vol II)

## I Introduction

A lot of ink has been spilt over the political importance of speech. But comparatively little has been written about the constructive political role of silence. Silence in public life has been discussed in the context of political philosophy and speech, but it is usually considered as a harmful outcome of silencing practices (e.g. Harris and McKinney 2021). In that context, the political importance of *breaking* silences has often been stressed (e.g. Medina 2023). However, the political importance of being able to stay silent has yet to be fully appreciated. In this article, we seek to redress this neglect. We propose a robust, democratic right to silence (RTS) in public life and argue that there are politically salient epistemic reasons for recognising that right.^1^

The RTS is well-known within the legal context (Kassin *et al*. 2019). ‘You have the right to remain silent’ is perhaps one of the most famous statements in the English language. It is part of the so-called ‘Miranda warning’ that US law enforcement officials must recite when they arrest someone, which is meant to inform the arrested person of their constitutional right against self-incrimination and to legal counsel. Yet, the limited legal RTS protected in some polities is too narrow and weak to protect the epistemic and other positive functions of silence in a liberal democracy. Indeed, even in the USA, where an apparently well-articulated legal framework enshrines the RTS, it is less well-protected than it might seem (Ainsworth 2012). Meanwhile, the right is practically non-existent in other liberal-democratic jurisdictions, such as the UK (Gardbaum 2021).

In this paper, we defend the need for a democratic RTS. A right can be defined as the entitlement to (not) perform a given action or be in a particular state or situation (Wenar 2020). Accordingly, we can broadly define the RTS as the entitlement to be silent, that is, to not speak *tout court* or about something in particular. This right has important implications for several core democratic rights and values, including political participation, equality, free speech, privacy, and knowledge.

As that suggests, silence and the right to it operate across many different registers, including the political, the moral, and the epistemic. In this paper, we specifically aim highlight the epistemic benefits of the RTS in democratic public life. Drawing on social epistemology, political philosophy, and rights theory, we will argue that the RTS provides two epistemic benefits.^2^ The first is that, if the RTS is recognised, we can avoid the epistemic harm that may be caused when an individual is compelled to lie in public. Such lies can jeopardise the individual’s credibility, and they can also have broader, collective implications, for example, by undermining the notion of truth. The second is that the RTS can protect the speaker from epistemic injustice. We will support these claims by examining two types of situations in which the RTS would help to avert epistemic harm and injustice. The first are situations where an individual might be compelled to lie publicly, particularly due to political, legal, and social pressure. The second are those in which an individual faces an audience that is pressuring them to speak but is likely to misconstrue what they say.

The RTS, as proposed, has its limits; it is not intended to protect people to speak against all pressures to speak. Nevertheless, it can have broad benefits. Specifically, the RTS could support the introduction of a series of norms and practices that could make people feel more comfortable with being silent in situations in which there is social pressure to speak. Elucidating the role of the RTS in the light of its epistemic impact has several benefits and contributes to two different literatures. Firstly, the literature on the RTS has tended to focus on the legal and moral implications of that right while saying little about its political and epistemic implications.^3^ Secondly, epistemological literature on the harms of compelled speech in social and political life has yet to give due consideration to how the RTS may help to protect against those harms (e.g. McKinney 2016; Pohlhaus 2020).^4^ This paper fills both those gaps.

The structure of this paper is the following. In Section II, we begin by defining silence and the right to it. We defend the need for a robust, democratic RTS that involves at least two duties toward rightsholders: a social duty and an epistemic duty. In Sections III and IV, we consider the epistemic value of the RTS by focusing on our main case studies. In Section V, we will conclude that the RTS plays a crucial role within the context of political speech, not only within the extent of public silencing practices but also for the epistemic role it plays in preventing forms of unjust compelled speech and epistemic injustices.

## II Outlining The Right To Silence

In this section, we unpack the RTS. We start by defining what kinds of silence it should protect and why these need protecting. We then draw on rights theory to sketch the shape of the right itself and articulate the duties it implies that other people have toward the rightsholder. Finally, we explain what kind of right the RTS is.

### II.1 What is silence?

Since silence can mean many things, we first need to clarify what it is that needs protecting. On our account, silence in the RTS refers to both the literal and metaphorical silences of a subject. Literal silence refers to an absence of speech from a subject. This can also be called outward silence to clarify that we are concerned with the silence of a subject rather than the silence around them (Degerman 2023).^5^ By contrast, metaphorical silence refers to not-speaking about something while speaking about something else. For example, when a defendant verbally invokes their Fifth Amendment rights when on the witness stand in a US court, they are speaking, but they might be said to be silent about the subject they were asked about in a metaphorical sense. The RTS encompasses both types of silence.

In this paper, we are mainly concerned with highlighting the need to protect literal silence. That is because such silence constitutes an essential mode for individuals to engage or disengage with and in public life (see Athanasiou 2017; Brito-Vieira 2019; Gray 2021). It is also because literal silence is particularly vulnerable to misinterpretation. To see why, we need further to distinguish between two kinds of literal silences: *eloquent silences* by which the subject intends to manifest a meaning, and *non-communicative silences* by which they do not (Tanesini 2018). Examples of the former include the silence of a wedding congregation when the priest asks if anyone objects to the marriage or the silent presence of protestors who have taped over their mouths in a public space. Examples of the latter include your silences while I am speaking or when you are considering what to say next. Importantly, non-communicative silences can still be taken as sources of information. For instance, I might take your silence while you are considering what to say as evidence of thought-fulness, but since you do not intend to convey thoughtfulness or any other meaning with your silence, it is not eloquent.

The communicative effect of both types of literal silence is radically context and audience-dependent (Gray 2019). Since literal silence does not involve words, its meaning must be inferred from what precedes, succeeds, or surrounds it. Therefore, literal silences are often deeply ambiguous and vulnerable to misinterpretation. Non-communicative silences might be especially so. A may have a very specific reason for keeping such a silence that they are not trying or even trying not to convey and, therefore, deliberately leave fewer clues and more ambiguity about its meaning. That non-communicative silence could be misinterpreted in two ways. First, you might recognise that I have a non-communicative reason for my silence but misinterpret what that reason is. For example, you might take it that the reason I respond to your question with silence is because I don’t know have an answer, when I was actually just concerned that you would react badly to my answer. Secondly, you might misconstrue my non-communicative silence as a communicative one. For example, you might interpret my silence in response to your question as an intentional snub even though I’m actually silent because I was thinking about something else and didn’t register your question.^6^ In either case, there is a correct interpretation of my non-communicative silence but there may be little evidence to allow you to arrive at it. That is frequently the case with public silences, making them highly difficult to interpret correctly. Yet, that difficulty does not prevent us from trying and getting it wrong, which, as we show later, can have serious consequences.

### II.2 What is the right to silence?

Having established the scope of the RTS, the next question is: What do we mean when we say that there is a *right* to silence and what does that right involve? We will answer this question using resources from rights theory.

The RTS can be conceptualised as a complex right, meaning that it consists of several so-called Hohfeldian incidents or what we will refer to as elements (see Wenar 2005).^7^ First, the RTS involves a *privilege element*. The individual has a privilege to be silent in public, which implies that they have no duty to speak. Secondly, it involves a *claim element*. The individual has a claim that others do not break her silence; this claim corresponds to duties for other people, which we shall elaborate on soon. Thirdly, it involves a *power element*. The individual has the power to waive others’ duty not to break her silence. Fourthly and finally, it involves an *immunity element*. Others cannot take away the individual’s privilege or claim upon them.

Duties are arguably at the heart of any right, as they stipulate how other people should treat the rightsholder (see Cruft 2013). Accordingly, we will emphasise the duties associated with the RTS. It is, therefore, worth explicating the kind of claim right the RTS involves and gives rise to those duties. The claim element of the RTS is one against others breaking the rightsholder’s silence. In the first instance, this is a claim against the interference of others rather than a claim that others should provide something like a service or a good. Thus, the claim element of the RTS can be understood as a negative claim right. That contrasts with a claim for the provision of goods and services, which is a positive claim right (Jones 1994: 15). However, since the claim element of the RTS and its implied duties are likely to be effective only if certain goods and services are in place, it may imply a positive claim as well.

That arguably holds about most, if not all, negative claim rights. Nevertheless, the distinction is helpful. Firstly, it clarifies that the RTS begins with a negative claim on others. Whatever positive claims might follow from that initial claim depends on whether others are responsive to that claim and, if not, what could facilitate greater responsiveness. Secondly, it supports our paper’s focus on individual and collective duties toward silent individuals, i.e. on moral and social rather than legal and institutional reforms.

Let us now spell out the duties implied by the RTS. The primary duty implied by the claim right of the RTS is a duty not to break the individual’s silence. To clarify what it involves and why it is needed, it can be divided into two sub-duties: a *social duty* and an *epistemic duty*. Each sub-duty corresponds to one way in which silence can be broken and is grounded in the ambiguity of silence discussed in the previous subsection.

The first and most obvious way silence can be broken is by making the silent person speak. This can be and often is accomplished through different forms of social pressure. We might generally think ourselves justified in exerting such pressure. However, our basis for thinking this is often weak because of the nature of silence, which means we cannot access the reasons why the silent person is not speaking. They may have ethical reasons for being silent that outweigh the good we think would be gained by breaking their silence, they may be pursuing the same good as us but through means they cannot tell us about, they agree with our goal but think the speech we demand of them would be an ineffective means of achieving it or they may have been intimated into silence. In any of those cases, pressuring the individual to break silence may cause harm and injustice. This motivates the *social duty* of the RTS, which is a duty to avoid pressuring the rightsholder to break their silence publicly. Performing this duty may involve both a negative obligation to avoid creating such pressure and a positive obligation to relieve pressure created by others.

The second, subtler way an individual’s silence can be broken is by others imposing an interpretation on it. Our collective hermeneutical resources are replete with tools for extracting information from someone’s silence, even when its meaning is ambiguous. These resources can range from interpretive schemas based on the personal experience of the interpreter—e.g. ‘When I am silent in a conversation, it is usually because I am afraid to speak up, so that must be why he is silent now’—and folk beliefs—e.g.‘he would not be silent if he did not have something to hide’—to social and psychological theories—e.g. ‘silence is rooted in stigma and fear’—and political taglines—e.g. ‘silence is violence’ (see e.g. Kenny 2018: ch. 1; see also Lackey 2018). These interpretations are not only liable to be mistaken since they do not take the actual intentions and testimony of the silent person into account, but, especially if publicly disseminated, they may also be difficult for the silent person to correct and they may constitute obstacles to the person’s effective participation in public life. Again, that may cause harm and injustice. This motivates the *epistemic duty*, which is a duty to refrain from imposing interpretations on a person’s ambiguous silence.

It is worth elaborating briefly on what that entails. After all, it might be impossible to avoid interpreting other people’s behaviours, and, if that is the case, as might seem plausible, the epistemic duty is impracticable. But the epistemic duty is not meant to prohibit our automatic interpretations of silence, though it could end up regulating them indirectly. Rather, it is meant to prohibit us from consciously accepting *unjustified* interpretations and sharing them in public in the absence of good evidence, and, more positively, to commit us to reflecting on the accuracy on our interpretations.^8^ Thus, we take the act of *imposing* an interpretation to be a more reflective and, potentially, interpersonal act than the act of merely interpreting.

The RTS, its claims and duties, and the other elements that constitute it should be understood as qualified. They must be weighed against other rights and duties in their implementation. Some rights have higher priority than others. For instance, my right to silence has a lower priority than someone’s right to life. So, if I could directly prevent someone’s imminent death by speaking, then my right to silence would be suspended in that situation. Putting it more generally, much like the right to free speech is often appropriately limited by considerations of the harms of speech, the RTS is limited by considerations of the harms of silence. Building on that parallel, we might say that a person has no right to stay silent about a fire in a movie theatre. Nevertheless, to paraphrase Leif Wenar (2020), while such qualifications ‘carve the contours’ of a right, ‘they do not affect its basic shape’.

We hope this analysis and argument for the RTS can provide a crucial starting point for weighing it against other rights. Recognising the RTS will demand an articulation of its limits as well. However, doing so is beyond the scope of the present paper. Since philosophical scholarship and political discourse tend to focus on the harms of silence and the duty to speak, it is more urgent and appropriate to begin articulating the value of protecting the RTS, as we do here.

### II.3 What kind of right is the right to silence?

The next step is to identify the kind of right that the RTS is. We approach the RTS as a ‘manifesto right’ (Feinberg 1973: 67), that is, as a right that *should* be recognised. We focus on providing epistemic arguments for its recognition and proposing some moral-epistemic reforms that its implementation demands.^9^

We are, hence, not arguing that it is already a legal right. It may be possible to argue that there is a legal basis for the RTS, but that is not our goal here. Neither are we arguing for the introduction of legal protections for the RTS. While such protections are likely needed, we are not yet sure how they should be framed.

Our focus on epistemic arguments for the RTS might raise the question of whether the RTS should be understood as an epistemic right. On Lani Watson’s (2021a) account, epistemic rights are complex rights that pertain to epistemic goods. They include rights such as the right to know, the right to information, and the right to understand; notably, Watson (2018) even suggests in passing that the narrower RTS in criminal proceedings is an epistemic right.^10^ Our subsequent arguments indicate that the RTS is *supportive* of epistemic rights so understood but is not itself an epistemic right. A right can be considered supportive of another right in two different ways. A right is *strongly supportive* of another right when the first right is necessary for the effective implementation of the second; it is *weakly supportive* of another right when the first is useful but not necessary for the effective implementation of the second (Nickel 2008). As we shall see, the RTS supports people’s ability to preserve knowledge, so the RTS is at least weakly supportive of epistemic rights. It could even be argued that the RTS is strongly supportive of epistemic rights insofar as without some degree of recognition of that right, epistemic rights are vulnerable to arbitrary violations.

However, categorising the RTS as an epistemic right would be a mistake because its scope extends beyond the epistemic into the moral and political. Recall the epigraph, which Creon utters in response to Oedipus forceful interrogation. They could read as implying that without the RTS, all other rights are thrown into question. That is hyperbolic, to be sure, but the RTS could be seen as strongly supportive of some core democratic rights, such as the right to free speech. Moreover, what we say or do not say is evidently not just of epistemic relevance. Our speech shapes the gamut of our public and, indeed, our private lives. We have, therefore, chosen to describe the RTS as a *democratic* right. By that, we mean to emphasise that the RTS justified by a broad set of epistemic, moral, and political values that are integral to the healthy functioning of a democracy.^11^ Across the following sections, we stress the epistemic justifications for the RTS, particularly those related to the epistemic agency and the right to knowledge, which are intrinsically and instrumentally crucial to a well-functioning democracy (see e.g. D’Agostini 2021a; Watson 2018).

## III The Epistemic Value Of The Right To Silence: Forced And Compelled Lies

The first reason the RTS is epistemically valuable is that it offers protection against the epistemic harm of forced and compelled public lies. It does this by relieving the individual of the pressure to speak under conditions in which they may feel compelled to lie publicly. This section will consider two kinds of cases: forced public confessions and compelled public lies. In both cases, we will see that breaking silence can lead to epistemic harm, facilitating the dissemination of false information.

### III.1 Forced public confessions and epistemic harm

Many factors in public and private life can pressure an individual into saying things they know to be false. For example, oppressive governments have been known to use threats and violence to force individuals to publicly confess to crimes they have not committed.^12^ The most famous instance of this is perhaps the Moscow Show Trials, in which 16 influential members of the Communist Party publicly confessed to being part of a terrorist group plotting to assassinate Stalin and other party leaders. The plot and the existence of the terrorist group were fabricated, the only evidence presented by the prosecution was the confessions of the defendants, and those confessions were extracted during interrogations by torturing the defendants and threatening their family members. After publicly repeating these confessions during the show trial, the 16 defendants were executed (Decker 2019).

Forced public confessions are not isolated to totalitarian systems. Sometimes, they occur in democratic countries, too (see Lackey 2021 and McKinney 2016), and they can serve in both totalitarian and democratic contexts. Obvious functions include deterring others from committing similar acts and preserving the political status quo. Others are epistemic, such as producing and disseminating politically useful falsehoods and, thereby, cause epistemic harm. As the sociologist Stephanie Decker (2019) observes, the purpose of the public confessions in the Moscow Show Trials was to ‘introduce alternative realities’, including facts about the particularities of the case—such as that the accused were part of a terrorist group—but also about more general matters—such as the type of threats that the Soviet Union faced. She also argues that these acts are effective in disseminating these ‘alternative realities’ partly because the confessions are uttered by the accused themselves. Insofar as forced confessions obscure the truth, we can say they are epistemically harmful. Furthermore, insofar as forced confessions cause epistemic harm because they are uttered by the accused, we can say that *ceteris paribus*, it would have been better if they could have kept silent. Accordingly, exercising the twin duties of the RTS—i.e. the social duty to avoid pressuring that the rightsholder to break silence and the epistemic duty to avoid imposing an interpretation on ambiguous silences of the rights holder—can provide an epistemic benefit: avoiding the dissemination of falsities and compromising the epistemic status of the information regarding the fact(s) considered.

Two concerns might arise about the relevance of this example. First, since the example relates to speech in the context of a trial, even if it is a sham trial, it may seem to speak more to the value of the narrower legal right to remain silent than the broader democratic RTS that we defend here. Perhaps it does. But although show trials take the form of legal proceedings, they are public events intended to influence people far beyond the courtroom. At the very least, show trials can be understood as a bridging case between the legal and the political, and they reveal some of the worst potential epistemic harms of compelled public lying. Moreover, the falsehoods produced and disseminated through forced public confessions—a kind of silence-breaking—might be used for political purposes or even to challenge the very notion of truth within the political sphere. That clearly has political implications since the concept of truth underpins many of the norms and rules—both formal and informal—that constitute the political sphere (D’Agostini 2021a). Hence, silence-breaking and the falsehoods it may disseminate are politically relevant.

Secondly, it might be observed that the absence of the RTS, narrow or broad, is one the lesser of the problems faced by someone subject to a show trial; the more urgent problem is that they are citizens of a state that uses lies, threats, and violence against its citizens with impunity. While that might be true, our point here is to illustrate that, regardless of the many other problems that facilitate forced public confessions, a robust RTS that leads people to adhere to the social and epistemic duties could protect against epistemic harms caused by a person having to affirm publicly what they know to be false.

These epistemic harms do not only concern the harmed person, as we will discuss in detail in the next section, but also the publics in which the information of forced confessions is disseminated.

### III.2 Compelled lies and epistemic harm

Individuals in liberal democratic societies are unlikely to find themselves subject to the sort of brutal pressure to lie publicly experienced by the victims of the Moscow Show Trials. But they are subject to other factors that might compel them to do so. The most salient force of this kind is social pressure.

John Stuart Mill famously worried about the epistemic harms of social pressure in articulating the principle of free discussion in *On Liberty* (Mill 2003 [1859]; see McLeod 2021). He observed it could be more effective than government intervention in enforcing conformity and suppressing dissent, and, hence, in obstructing the discovery of truths and falsehoods that occurs through free discussion: ‘Society [is capable of] a social tyranny more formidable than many kinds of political oppression, since, though not usually upheld by such extreme penalties, it leaves fewer means of escape, penetrating much more deeply into the details of life, and enslaving the soul itself ‘ (Mill 2003: 76). That remains the case in advanced liberal democracies, where new technologies such as smartphones and social media mean that social pressure is perhaps more pervasive than ever.

As in much literature on political and free speech today, silence features in Mill’s discussion only as an epistemic harm. Social pressure, he notes, is prone to silencing those who hold unpopular opinions, true or false, and, hence, deprive people in general of the benefits of acquiring new truths or clarifying extant truths in the light of falsehoods. Notably, however, he acknowledges that social pressure can also compel individuals to publicly avow what they regard as falsehoods. Mill says it breeds ‘mere conformers to commonplace, or time-servers for truth, whose arguments on all great subjects are meant for their hearers, and are not those which have convinced themselves’ (p. 101). His disapprobation of such compelled public lies suggests that not all publicly uttered falsehoods are epistemically beneficial. We might recall that one of Mill’s key arguments for free discussion is that even the airing of false opinions can lead to a better understanding of the truth. But he does not seem to think that public lies that confirm prevailing opinion offer this benefit. Considering his broader analysis, one plausible reason is that when an individual publicly avows a prevailing opinion for non-epistemic reasons, they cause epistemic harm by putting truth further out of reach for everyone (see also Shiffrin 2014: 142–4).

Nevertheless, Mill fails to recognise that when social pressure would make people lie, silence might be epistemically preferable to speaking. Instead, he suggests that there is little difference in the epistemic consequences of responding to social pressure by publicly conforming or responding by being silent on matters on which one’s views are controversial. Again, we would suggest that equating the two is a mistake, for while an individual’s silence might not arrest the spread of a falsehood, it at least does not amplify it.

Mill’s project is more ambitious and optimistic than ours. His critical analysis highlights that social pressure results in non-epistemic values that conflict with epistemic values (cf. Rooney 2017). This leads to a familiar dilemma: either I speak the truth and harm myself or lie and harm the truth. When faced with those options, people are likely to choose the former. Mill’s proposal for solving this problem is effectively to remove the social hazards of dissent. He argues that this should involve prohibitions on responding to unpopular opinions with anger, moral condemnation, or social exclusion (2003: 143). We agree that free discussion is epistemically crucial, and democratic citizens and institutions should seek to defend extant and erect new protections against social pressure that might lead individuals either to lie or remain silent for fear of the consequences of saying what they think. However, it does not seem feasible or even desirable to altogether remove social sanctions against dissent. Meanwhile, current norms and laws protecting freedom of expression are insufficient to safeguard everyone from social pressure that might compel them to lie.

The RTS, then, constitutes a complementary means of reducing epistemic harm that can arise when social pressure compels an individual to choose non-epistemic values over epistemic ones. We have argued that in situations where the options are to tell a lie or to be silent, it is, *ceteris paribus*, epistemically preferable for an individual to remain silent than to throw their epistemic weight behind a lie. If the RTS is sufficiently strong, they can, thus, choose a third option that resolves the dilemma, in which they will neither harm themselves nor the truth.

Silence is only a viable escape from the rock of social hazard and the hard place of epistemic harm so long as its meaning is ambiguous. But, as mentioned in the previous section, our collective hermeneutical toolboxes contain many resources capable of imposing meaning on silence regardless of why an individual is silent or if their silence had communicative intent. One commonly used phrase in public life that can be deployed to this end is ‘silence is violence’. There is no doubt that silence can be violence, but then so can speech. Indeed, when some people have been accused of doing violence with their silence, there seems to have been little regard for the harm their speech could have done to those close to them. That was the case during the early stages of the Russian invasion of Ukraine, for example, when some prominent Russian expatriates were pressured to break their silence about the invasion by their employers. When the meaning of silence is predetermined in such a fashion, the dilemma might be transformed into a trilemma: either I speak the truth and harm myself, I stay silent and harm myself, or I lie and harm the truth.^13^ Either way, lying again seems the more likely choice. This is why both the social and the epistemic duty are crucial. Letting people be silent and thereby avoid lying is of little epistemic value in public life if we are going to fill their silence with our own lies, untruths, or half-truths.

We have focused here only on how the RTS can help to avert the epistemic harm of forced and socially compelled lies. Yet, it is worth noting that the RTS help to tackle other forms of lies and manipulation in politics and public life more broadly that arise due to social pressure and cause widespread epistemic harm. Adam Gibbons (2023), for example, has argued the pressure to appear politically well-informed incentivises people to utter political bullshit. A correct application of the RTS in which rightsholders are able to assert that right and others exercise social and epistemic duties towards them could prevent the dissemination of and perhaps even decrease the amount of compelled bullshit, compelled half-truths, and related phenomena that might affect the public life and undermine democratic rights and values.^14^

### III.3 Two objections

Before examining another epistemic benefit of the RTS, we want to address two potential objections to our proposal. First, a critic might argue that—as in the case of forced confessions—the RTS would not address the real problem, which is that individuals find themselves in circumstances that force them to prioritise non-epistemic values over epistemic values. Those circumstances themselves seem both morally and epistemically unjust. If we want to reliably prevent the epistemic harm of people being compelled to lie publicly, we would be well-advised to treat the causes of those circumstances. Meanwhile, the proposal to recognise the RTS might seem only to treat the symptoms or the surface-level causes of epistemic harm. However, even if that were true, it does not contradict our argument that the RTS is epistemically valuable because it would prevent some instances of compelled lying and, thereby, the epistemic harm that stems from them. This can hold regardless of whether there are other and perhaps better means of achieving the same end.

A second and perhaps more pressing concern is whether the RTS is redundant, given that existing rights seem to cover similar territory. Saliently, the right to free speech, it might be pointed out, already seems to protect against compelled speech. For example, in the USA, several Supreme Court decisions have affirmed that the First Amendment ‘prohibits the government from telling people what they must say’.^15^ Yet, as illustrated by the example of the Russian expatriates being pressured to break their silence on the invasion of Ukraine, such rights offer insufficient protection against the social forces that can compel us to break silence and, potentially, lie in public. Recognising the RTS can complement the protections provided by extant rights by offering additional, principled reasons for people to allow rightsholders their silence and clarifying the duties that the former owe to the latter. Furthermore, the fact that extant rights and laws overlap with the RTS does not weaken the case for recognising the latter. On the contrary, as we noted earlier, the RTS can be understood as supportive of other rights. That includes the right to free speech which some have argued is an empty right without the RTS to complement it (Henley 2010).^16^

## IV The Epistemic Value Of The Right To Silence: Problematic Inclusion And Epistemic Injustice

So far, we have argued that the RTS is epistemically valuable because it allows individuals to avoid situations that might otherwise have compelled them to lie publicly, thereby causing epistemic harm. This value is epistemic in the traditional sense. That is, it is valuable by virtue of its role in preventing the obstruction of knowledge and protecting of truth, and it is so regardless of the other sorts of benefits it might confer on individuals or groups. Yet, our discussion has already gestured at another key value of the RTS. After all, when someone is forced to lie, it is not just collective knowledge that is harmed. The individual who finds themselves subject to the compulsion is also profoundly harmed in other ways. For example, the hijacking of their voice to achieve ends that are not their own violates their political and personal autonomy. Some of those harms can also be understood as epistemic insofar as they impact the speaker not just as a moral or political subject but as a *knower*. That is, they may constitute an epistemic injustice (Fricker 2007). The consideration of the RTS in these contexts provides a unique advantage to these debates, as it focuses on cases in which silence might be beneficial. Often, in the epistemic injustice literature silence is considered only in relation to silencing someone in virtue of not recognising their epistemic authority, as for example in some cases of testimonial injustice. Here, we will show that breaking silence—and thus not respecting RTS—might lead equally to forms of epistemic injustices.

The Moscow Show Trials illustrate this well. The accused were used to create an alternative reality. But it was not just their gruesome fate or abused bodies that were used to this end. Their testimony publicly affirming that reality was considered crucial and, thus, extracted from them, too. So, besides being deprived of political rights, personal autonomy, and bodily integrity, the epistemic agency of the accused—their capacity to have and share knowledge— was also violated. In other words, they were subjected to epistemic injustice. By helping people avoid situations in which they might be forced to lie, the RTS may, thus, also help people avoid epistemic injustice.

The second value of the RTS is its capacity to protect individuals and groups against epistemic injustice. However, as we will show in this section, this goes beyond protections against the epistemic harms of compelled public lying. It extends to what is an equally, if not more, common kind of epistemically problematic situation, namely, the kind in which an individual faces an audience that is pressuring them to speak but is likely to misconstrue what they say.

Much of the epistemic injustice literature has focused on how marginalised individuals are excluded from knowledge production, primarily in terms of how they are denied credibility—i.e. testimonial injustice—or how they are deprived of the resources that would allow them to participate effectively in the production of knowledge—i.e. hermeneutical injustice (see Kidd *et al*. 2017). Unsurprisingly, silence is a prominent theme in this literature. Silence is a symptom of epistemic injustice and a cause that perpetuates it (e.g. Dotson 2011). It is also something that the inclusion of marginalised people in the spaces and processes of knowledge production is meant to break (e.g. Rose 2017).

More recently, some scholars have started to problematise that emphasis on inclusion. For instance, Gaile Pohlhaus (2020) argues that epistemic inclusions can be as pernicious as epistemic exclusions. One reason is that when marginalised group members are deliberately included in knowledge production processes, their contributions are often distorted.

That is the case in what Emmalon Davis (2016) calls compulsory representation. This is when a marginalised individual is included in an epistemic exchange only because they are regarded as representative of and possessing special knowledge about their community. Unlike the standard types of epistemic injustice, which involve an identity-prejudicial credibility deficit, Davis argues that compulsory representation involves an identity-prejudicial credibility excess, in which an individual is afforded more credibility than is warranted because of their social identity. Credibility excess may seem like a boon, and sometimes it can be. But, in compulsory representation, at least, it is epistemically harmful. When dominantly situated groups call upon a marginalised individual as a spokesperson, their interests often extend only to what is unique about that individual’s community while disregarding what the individual may have to offer as a unique subject. The individual, meanwhile, may be aware of their ‘host’ expects of them as well as the potential ramifications that their testimony could have for those in the community they are taken to represent. And, so, they might adapt their testimony, perhaps by suppressing the particularities of their own experience while trying to present a picture that reflects the average experience in their community as they see it. Hence, the individual’s epistemic agency remains highly circumscribed, which their formal inclusion is likely to obscure.

Compulsory representation does not just threaten the epistemic agency of the speaker but also that of the group they are taken to speak for. Sometimes, political processes may require spokespeople to represent a wider community, perhaps because various constraints make it unfeasible for all community members to participate. Townsend & Lupin (2021) argue that for someone to count as a spokesperson in such cases, they must fulfil three criteria. A spokesperson must be authorised to speak by that group, she should understand that she is speaking for it, and the relevant audience should take her to be speaking for the group (pp. 586–8). In compulsory representation, however, often only the second and the third conditions are fulfilled—sometimes only the third.^17^ Nevertheless, as long as a member of the community participates in, for example, a policy consultation, Townend and Lupin note that the officials may be satisfied that the community’s voice has been heard and end the process, meaning that the community has lost the opportunity to be heard (p. 590). Thereby, compulsory representation can diminish the epistemic agency of entire communities.

While these scholars take a more nuanced view of the role of inclusion in addressing epistemic injustice, they still thematise silence only as a negative phenomenon, contrasting it against the good of speech. Yet, they highlight instances where the pressure to produce speech under hostile conditions and the resulting speech is epistemically harmful. In compulsory representation, the audience pressures an individual to speak; then, it extrapolates what that individual says to their community, regardless of whether they intend to represent anyone but themselves. Whatever the individual says may, thus, affect others in their community.

Some who face compulsory representation may reconcile themselves to the epistemic burden of being a spokesperson of their community, albeit one unchosen by the community itself. They answer questions from those who want to be educated about their community and try to become as knowledgeable as they are assumed to be (Davis 2016: 491–3). However, the epistemic issues associated with compulsory representation do not disappear simply because the individual deliberately assumes that burden. The individual has still had to truncate their epistemic subjectivity. And, despite their best efforts, they are still an unauthorised spokesperson, and their participation may still mean that others lose their opportunity to speak. Moreover, since their epistemic labour is likely to be ‘unrecognized, uncompensated, emotionally taxing’ and is, at least sometimes, extracted under coercive conditions, it arguably involves another epistemic injustice too, namely, epistemic exploitation (Berenstain 2016: 569; see also Davis 2016: 492). More speech may, hence, mean more injustice in circumstances such as these (see also Pohlhaus 2020: 245).

Others who face the epistemic injustice of compulsory representation opt for a different response, which is more symmetrical to the problem. Faced with an audience that both demands that they speak yet will reliably misconstrue what they say, some people choose silence. We find several examples of this in Elizabeth Ganter’s book *Reluctant Representatives* (2016), a study of Aboriginal and Torres Strait Islanders working as public servants in Australia and their experiences of acting as representatives of their community. As the book’s title suggests, many of those who participated were reluctant to take up the role of representative; indeed, some chose to reject it.

Consider the following extract from an interview with an

Aboriginal public servant identified as Jay.

[ Jay: ] You go to workshops… every Wednesday morning… [T]he word Indigenous comes up and everybody looks straight at me…

[Ganter: ] How *do* you respond?

[ Jay: ] Oh, I’m at the stage now where I don’t. So they’ll look at me and I’ll look back at them with a blank face. (Ganter 2016: 99)

Fed up with the expectation to speak on behalf of Aboriginal people, Jay responds with defiant silence.

Another study participant, Bob, related a similar experience in a manner that suggests an awareness of the potential epistemic harm if he succumbed to the pressure to speak on behalf of his community. Having been invited to participate in an Aboriginal staff forum in his department, Bob worried that his department would use his participation to falsely claim substantive engagement with the Aboriginal community. In the end, he did participate, but, in Ganter’s words, he ‘had adopted a tactical reserve, and was uncommunicative when invited to substitute for those whom he felt should be heard firsthand’ (p. 100).

Drawing on Dotson’s (2011) concept of testimonial smothering, Townsend & Lupin (2021) interpret these two accounts as evidence that Jay and Bob are the victims of silencing. Having realised that their speech would be taken as representative of Aboriginal people in general, Jay and Bob decided to respond with silence. We think this is a mistake that obscures the epistemic benefits of silence and the right to it.

It is unjust that Jay and Bob are expected to represent others who share a social identity, especially despite their explicit efforts to reject that expectation. In that sense, they are victims. Yet, to reduce Jay and Bob’s silence to a symptom of victimhood and a case of testimonial smothering is to elide the epistemic agency that it involves. Even though their silence is a response to unjust circumstances, it is also an act of epistemic resistance. It resists efforts to extract knowledge from them, knowledge that risks being distorted and used to leave the rest of their community voiceless. Instructively, Ganter herself describes Jay’s silence as resistance: ‘Jay could only resist the subtle pressure to substitute for the absent by arranging a ‘blank face” (2016: 99).

As mentioned, some political theorists have called attention to the potential political power of silence. Notably, Vincent Jungkunz (2011) suggests that perhaps ‘the most fundamental way in which silence can be deployed politically is to, in an untimely manner, refuse interpellation’. This is what Jay and Bob did; they refused the request of an overbearing audience that could not be trusted to understand them correctly. But Jungkunz and others have overlooked the epistemic dimension that such silent refusal often has. By remaining silent in the face of a request for information, Jay and Bob frustrated an epistemic process that is, arguably, intrinsically unjust. If allowed to continue unabated, that process would likely perpetuate and perpetrate epistemic injustices, such as compulsory representation and epistemic exploitation. To the extent that their silence has political power, it is, then, linked to how it affects the flow of knowledge.

All this speaks to the broader argument that the RTS is epistemically valuable in democratic public life. Compulsory representation and epistemic exploitation are just some of the epistemic injustices that loom when an individual faces the pressure to speak in circumstances when their audience would reliably misunderstand them irrespective of whether that is in a formal political, civic, cultural, or professional context. While such pressures may confront anyone, including someone in a position of power, they are likely to be more severe for marginalised individuals. That is because marginalised individuals are likely to face other epistemic disadvantages and injustices too, such as lack of testimonial credibility or hermeneutical resources. Consequently, they may feel a stronger drive to speak when asked, even when speaking might be counterproductive or harmful to themselves or others. So, while silence can offer protection for anyone facing the pressure to speak in circumstances where this could cause epistemic harm, it may be a particularly important resource to marginalised individuals.

To conclude, we have considered this second type of case to show that failing to recognise and respect the RTS can lead to epistemic injustice. This might happen when the rightsholder is pressured to break their silence, such as with compelled lies or compulsory representation. It can also happen when the hermeneutical tools deployed deprive the intended and political meaning of someone’s silence, as in the last case considered. Thus, breaking their silence either by forcing them to speak or by imposing an interpretation on their silence compromises the status of the knower, who may end up lying or failing to be recognised as a knower.

### IV.1 A final objection

A sceptical reader might acknowledge that silence and, by extension, the right to it can offer protection from some instances of epistemic injustice. However, they might also remark that Jay and Bob could deploy silence to this end without the sort of robust RTS that we have outlined. While that seems right, it is noteworthy that Ganter introduces her study with a dilemma that many of her participants faced: to stay silent in defiance of their work description or superiors and, thus, risk professional consequences, or to speak and, thus, risk being misunderstood and having one’s words misused (Ganter 2016: 3). If Jay and Bob feared the professional consequences of their silence, it was not enough to deter them.

However, for many people who face such dilemmas in their public life, the consequences of silence may be significant enough to outweigh the epistemic reasons for silence. For them, a robust RTS that provides the conceptual tools to claim the ambiguity of silence for oneself and defend it from others could be transformative. This could be done, as we have suggested, by acknowledging and defending the epistemic dimension of that right and considering the epistemic benefits it implies. Moreover, these considerations contribute to hermeneutical tools that citizens can use to claim and protect their RTS. We argue, thus, that the awareness of all the dimensions implied by the RTS, particularly the epistemic one, brings epistemic tools to those exercising it and supports the pursuit of epistemic justice.

## V Conclusion

The epistemic importance of the RTS within public life demands more philosophical attention. Broadly defined, the RTS is the entitlement not to speak in public life. That right involves a negative claim on other people, namely, that they do not break the individual’s silence. That claim implies two duties, a social and an epistemic one. The first implies that one should not pressure right-holders to break their silence and the second that one should not impose interpretations on ambiguous silences.

In this paper, we have sought to demonstrate the value of the RTS within public life. In particular, we have argued that the RTS can provide two epistemic benefits.

The first benefit is that it offers protection against the epistemic harm of public lies. More specifically, it relieves individuals of pressure to speak under conditions in which they may feel compelled to lie publicly, either within political and jurisdictional contexts or because of social pressure. The epistemic harm that follows from such cases is the undermining of truth and knowledge, which in turn may lead to further epistemic and non-epistemic harms because of the structural role of truth at different levels (D’Agostini 2021). This also illustrates the political impact that the RTS can have: By protecting the RTS, there is the possibility to protect truth and its political role further. Accordingly, recognising the RTS can prevent such epistemic harm by creating an environment where silence is respected and does not have to be broken.

The second epistemic benefit of the RTS is that it protects people against some epistemic injustices. Many epistemic injustices can follow from breaking someone’s silence, but here we focused on two. The first is that, as in the case of compelled lies, if one is forced to lie, their authority as knowers is compromised. The second and more original context is problematic inclusion and compulsory representation. In these cases, representatives of communities can be forced to speak on behalf of others while not having the authority and the willingness to do so. This can put them in the position of being given credibility excess, which is also a form of epistemic injustice.

We hope then that we have convinced you that although the RTS might not be quite so fundamental as Creon’s words suggests, it is nevertheless crucial to maintaining the epistemic integrity of a democracy and we, democrats, ought to recognise it.^18^

## Footnotes

1 We define public life broadly to encompass interactions with various publics, including political, civic, cultural, religious, and professional ones. This contrasts with private life, which encompasses interactions with family and friends as well as one’s thoughts. Democratic theorists inspired by, for example, Hannah Arendt’s (1998) or Jurgen Habermas’s (1999) respective theories of the public will find this definition too broad. However, our central arguments for the RTS are plausibly applicable to their narrower understandings of public life too. Moreover, the merit of starting with a broad operative definition of public life in outlining the RTS is that it allows us to indicate the broad potential relevance of that right.

2 By epistemic, we mean broadly those aspects that consider doxastic attitudes, such as beliefs, truth, and knowledge, in relation to their effect on social interactions and social systems. Accordingly, this paper aims to highlight the epistemic benefits of the RTS within the system of political and social interactions (Goldman and O’Connor 2021).

3 For example, in US legal theory, the RTS has often been justified in terms of protecting against compelled speech. However, the defenders of the RTS stress the moral consequences of compelled speech for the individual. As one scholar puts it: ‘the right to remain silent is grounded primarily in a desire to avoid the individual’s humiliation, demoralization, and cognitive dissonance that flows from being forced by the government to speak’ (Redish 2013: 151).

4 Our argument complements Lackey’s (2023) brief discussion of the role of the right to remain silent in protecting criminal defendants’ epistemic agency (pp. 50–1).

5 The case could be made that the RTS should encompass outer silences—such as the lack of obtrusive noise in the subject’s environment—as well (see Sim 2007), but that is not our aim here.

6 Thanks to Anthony Everett for pointing out this distinction to us.

7 This analytical framework was conceived by Wesley Hohfeld (1919) and is widely used in rights theory. We deploy Wenar’s (2005) refined version of this framework.

8 We thank one of the journal’s referee’s for encouraging us to make this point.

9 This effectively corresponds to what is usually called a moral right in contemporary rights theory (Wenar 2020). However, since moral reasons are often contrasted against epistemic reasons in philosophy of language and epistemology, we eschew the term moral right to avoid confusion.

10 Watson’s (2021a) concept of epistemic rights can be compared to D’Agostini’s (2021a) concept of alethic rights. Their debate suggests that, despite important differences, the two concepts may be complementary (Watson 2021b; D’Agostini 2021b). Here, we prefer the epistemic rights concept because it resonates with our articulation of and arguments for the RTS.

11 This resonates with Brettschneider’s (2007) conceptualisation of democratic rights, though we are not committed only to the three values he outlines nor that all democracies are characterised by the same set of values.

12 Some argue that coerced speech should not be considered lying because an utterance must involve a freely chosen intention to be deceptive to count as a lie (e.g. Hawley 2018). Others disagree and argue that coerced statements are lies even though they do not involve deceptive intent (e.g. Sneddon 2021). We do not mean to take a stand on this debate here by using terms like compelled ‘lies’ and ‘lying’. We do so only to distinguish compelled speech, in which the intended meaning is false, from compelled speech more broadly, in which the intended meaning may be either true or false, instead of using more unwieldy phrases such as ‘compelled false speech’. However, we presume people in the kinds of cases discussed here want the meaning of their utterance to be taken as truth, because, otherwise, the real or perceived threat against them would be realised. So, in an important sense, they do intend to be deceptive, even if that intent is generated by coercion.

13 Notably, in US law, a foundational rationale for the Fifth Amendment and the right to remain silent was to guard against ‘the cruel trilemma’ that many people accused of a crime faced of being forced to choose between self-incrimination, perjury, or contempt of court if one refused to speak (Bentz 2012).

14 We owe this point to one of the journal’s referees.

15
Https://www.thefire.org/supreme-court/donald-h-rumsfeld-secretary-defense-et-al-v-forum-academic-and-institutional-rights

16 Another right closely related and plausibly supported by and supportive of the RTS is the right to privacy. For a relevant analysis of the democratic importance of privacy, see Lever (2006). Unpacking the complex relationship between these two rights is beyond the scope of this paper but will be an important next step in developing the RTS.

17 Townsend & Lupin (2021) mean something slightly different from Davis by ‘compulsory representation’. They distinguish between an individual being taken to speak as a group member and being taken to *speak for* a group. However, as they note, this is a subtle distinction, which does not affect our analysis.

18 We would like to thank Havi Carel, Ana-Maria Cretu, Anthony Everett, Kerrin Jacobs, Ross Pain, the journal’s anonymous referees, and audiences at Hokkaido University and the 2022 IRNEP Philosophy and Economics Conference for their helpful feedback on different versions of this paper. Dan Degerman’s work on this paper was funded by the Leverhulme Trust (ECF-2020-583), the Arts and Humanities Research Council (AH/Y001508/1), and the Wellcome Trust (226603/Z/22/Z).
